# An ecological model of vaginal dysbiosis provides new research leads

**DOI:** 10.1371/journal.pbio.3003604

**Published:** 2026-02-04

**Authors:** François Massol

**Affiliations:** Institut d’Ecologie et des Sciences de l’Environnement de Paris (UMR7618), Sorbonne Université, Université Paris Cité, Université Paris Est Créteil, CNRS, INRAE, IRD, Paris, France

## Abstract

While clinical features of bacterial vaginosis have been amply described, its underlying causes are still debated. This Primer explores a new ecological model published in PLOS Biology that provides a solid hypothesis corroborated by existing data, hinting at new prophylactic strategies.

## Teaser

While clinical features of bacterial vaginosis have been amply described, its underlying causes are still debated. A new ecological model published in *PLOS Biology* provides a solid hypothesis corroborated by existing data, hinting at new prophylactic strategies.

**Fig 1:**
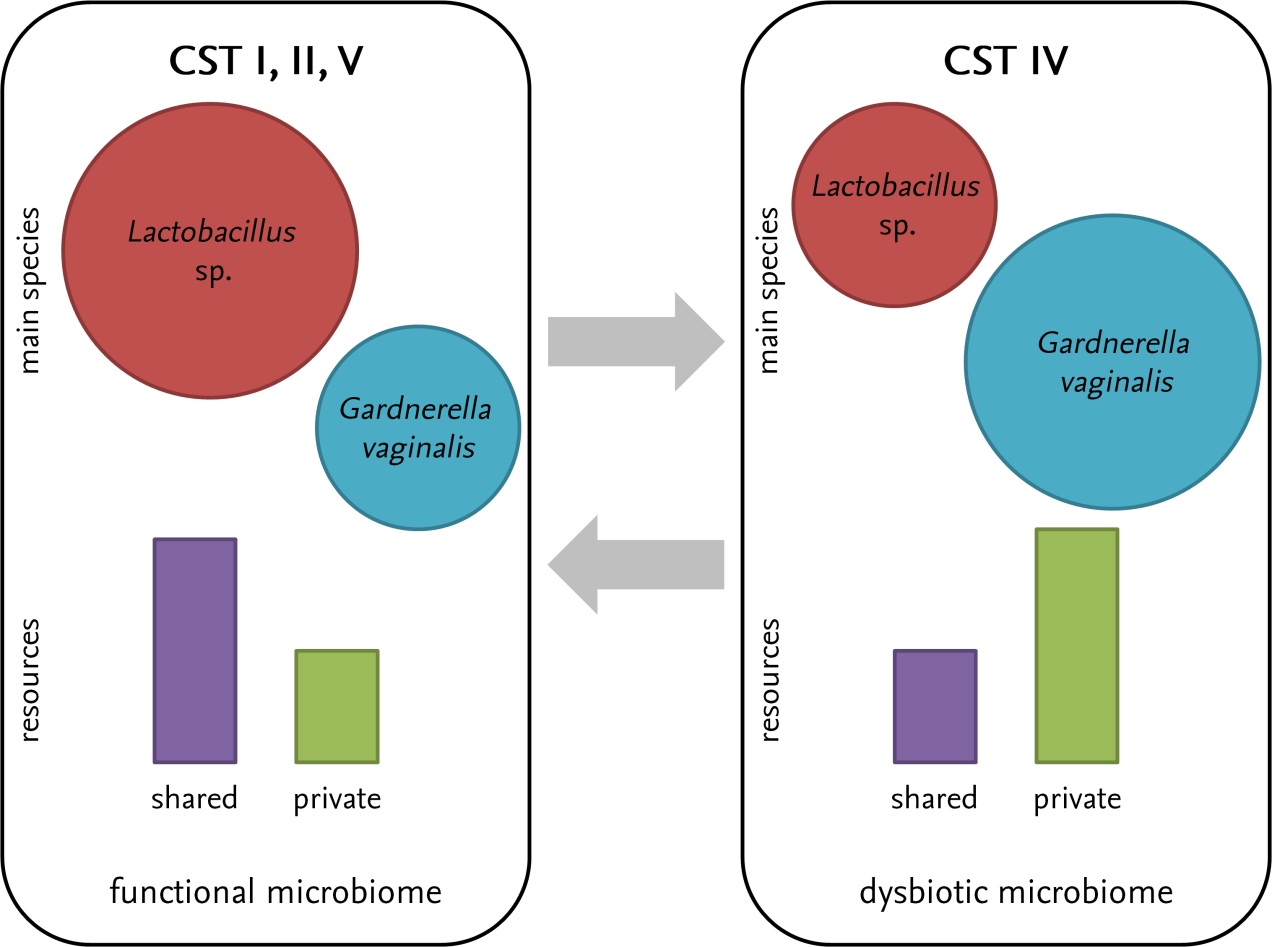
A schematic view of the vaginal dysbiosis problem. Absence of bacterial vaginosis is associated with functional microbiomes in which *Lactobacillus* sp. are dominant and shared resources (mostly glycogen) are the most abundant, mainly represented by community state types (CST) I, II, and V (left-hand side box). Dysbiosis, by contrast, is associated with CST IV in which bacteria like *Gardnerella vaginalis* are dominant and private resources (i.e., sialoglycans) are abundant (right-hand side box). Transitions between these two states are associated with changes in both microbial community states (relative abundances) and shared-to-private relative resource ratios.

Microbial ecology has proved to be fertile ground for testing ecological theories time and again [1], but it can also help in very practical ways—in the present case, by understanding the determinants of dysbiosis (i.e., altered functioning through altered composition) of the vaginal microbiome. Indeed, bacterial vaginosis (a bacterial infection of the vagina) is known to be strongly associated with some particular types of bacterial communities [2,3]. Basically, vaginal microbiota have been categorized into several community state types (CST), among which one such community type (CST IV) shows a very strong association with vaginosis. Why these CST arise, why these bacterial communities shift in response to their environment, and thus why some vaginal microbiota tend to favor the occurrence of bacterial vaginosis, can be studied in the light of ecological theory by focusing on the role of resource heterogeneity (Fig 1).

Studies on various types of microbial communities structured by different interactions—such as competitive [4], parasitic [5], and mutualistic [6]—show that heterogeneity in resource acquisition ability is very important for understanding how these communities change and develop over time, both in the short term and in the long run. Indeed, a cornerstone model of theoretical ecology has brought forth resource ratios as the main explanation of species niches based on the relative regeneration rates of limiting resources [7]. One of the most intense forms of heterogeneity in resource acquisition ability among species occurs when some resources become “private”, i.e., when only a particular species or genotype can access a critical resource. Empirical evidence on bacteria found within vaginal microbiomes suggests that such a privatization of resources might be occurring, as only a few bacterial species can make use of sialoglycans [8].

The newly published results from the mechanistic model of Kamiya and colleagues [9], backed up by existing data, indicate that resources available for bacteria can explain a large part of the variability of vaginal microbial community composition, which paves the way for novel means of prevention and treatment of bacterial vaginosis. More precisely, Kamiya and colleagues [9] propose a simple ecological model of resource competition between bacterial types. This model assumes that nondysbiotic microbial communities control the vaginal pH, and thus inhibit the growth of other bacterial types, while bacterial strains leading to vaginosis (CST IV) benefit from potential private resources. Succeeding at what it has been built for, this model qualitatively reproduces the possible states of vaginal microbiomes and predicts the occurrence of bistable outcomes, i.e., two alternative stable states more or less easily attainable based on initial conditions. However, the real feat comes from the derived predictions: based on their model, Kamiya and colleagues are able to formulate a predicted association between the proportion of resource supply that is private to CST IV and the occurrence of vaginal dysbiosis, with an associated predicted sharp transition between the two states. Using data from a cohort in which resource-associated metabolites and bacterial communities were assessed, Kamiya and colleagues [9] put their model to the test and the result seems quite positively conclusive. Indeed, when plotted against the productivities of shared and private resource metabolites, the state of the vaginal microbiome abruptly shifts above a certain proportion of private resources. Changes in shared and private resource relative production are also well associated with temporal changes in CST, thus supporting the expected dynamics of the model.

This study achieves an interesting result using remarkable methods—the proposed model is indeed using minimal ecological assumptions, and thus aims at maximal genericity—while at the same time providing an inspiring example of a study bridging many worlds at once (clinical microbiology, ecology, theory, and empirical data) through a careful use of statistics to connect model and data [10]. Kamiya and colleagues’ [9] study also paves the way for a variety of future research directions. On the practical side, leveraging the dependence on private resources of CST IV could be used for prophylaxis or as a component of therapies against bacterial vaginosis, much like the combination of para-aminobenzoic acid (pABA) deprivation and antimicrobial treatment has been advocated as a means to fight against the emergence of antimicrobial drug resistance of *Plasmodium chabaudi* in mouse models [4]. Both in vitro and in vivo tests of such prophylactic and therapeutic strategies should be envisaged, as the potential for unexpected interactions with other components of the vaginal microbiome is not negligible. On the more theoretical side, there is also room for the development of model refinements, such as the inclusion of different sources of vaginal colonization by bacteria, perturbations of vaginal pH or of the microbiota through collateral antibiotic effects, in order to encompass a wider variety of clinical situations, or even interactions between the immune system and the microbiome [11]. One could also endeavor to replace this model in the framework of host-microbe evolution in order to gain more insights regarding the potential directions of evolution of vaginal microbes in the face of changes in human diet, lifestyle, and antibiotic use, and how this evolution can interact with the classic typology of vaginal community types.

## Abbreviation:

CST: community state types;
pABA: para-aminobenzoic acid
